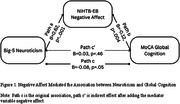# The Association Between Neuroticism and Global Cognition is Mediated by Negative Affect: Findings from the I‐CONECT Study with Socially Isolated Older Old

**DOI:** 10.1002/alz.087560

**Published:** 2025-01-09

**Authors:** Kexin Yu, Chao‐Yi Wu, Jennifer R. Gatchel, Hiroko H Dodge

**Affiliations:** ^1^ NIA‐Layton Aging & Alzheimer’s Disease Research Center, Oregon Health & Science University, Portland, OR USA; ^2^ Oregon Center for Aging & Technology (ORCATECH), Portland, OR USA; ^3^ Massachusetts General Hospital, Harvard Medical School, Boston, MA USA; ^4^ Baylor College of Medicine, Houston, TX USA

## Abstract

**Background:**

Personality traits are long‐standing characteristics of behavior and emotion. Personality might influence cognitive health in later life by affecting responses to stressful events and engagement in cognitively stimulating activities, such as interpersonal interaction. The current study examined associations between personality traits and cognition in late life, and potential mediation by positive and negative emotion, depression, and social connectedness.

**Method:**

We used baseline data from the Internet‐Based Conversational Engagement Clinical Trial (I‐CONECT) study. The I‐CONECT study recruited socially isolated older adults who were 75+ and were either cognitively unimpaired (CU) or had mild cognitive impairment (MCI). Big‐5 personality traits (neuroticism, extraversion, agreeableness, conscientiousness, and openness) were measured with the NEO‐FFI Personality Inventory. Global cognition was assessed with the Montreal Cognitive Assessment (MoCA); depressive symptoms with the 15‐item Geriatric Depression Scale, social connectedness with the 6‐item Lubben Social Network Scale (LSNS‐6), and emotional characteristics (negative affect, social satisfaction, and psychological wellbeing domains) with the NIH Toolbox‐emotion battery (NIHT‐EB). We ran general linear models to identify which Big‐5 personality traits were related to the MoCA score and to establish relationships between the Big‐5 personality trait and proposed mediators. Then, we ran mediation analyses. All analyses controlled for age, sex, and education. Sensitivity analysis included MCI status as a covariate.

**Result:**

The analytical sample included 146 participants (female 69.2%, mean age 81.1 years, mean education 15.2 years, MCI 56.2%). Out of the five personality traits, only Neuroticism was related to MoCA (Path c, β = ‐0.08, p = 0.05). The mediation analysis showed that negative affect mediated the association between Neuroticism and MoCA (coefficients in Figure 1). Controlling for MCI did not change the model results. All the other mediators, including GDS‐15, LSNS‐6, and social satisfaction and psychological wellbeing did not mediate the relationship.

**Conclusion:**

The neuroticism trait in socially isolated older adults was associated with worse global cognition, and this relationship was mediated by symptoms of negative affect. Results suggest that the NIHTB‐EB may capture symptoms of negative affect distinct from the GDS‐15. Behavioral health interventions aimed at delaying cognitive decline could consider addressing negative affect for individuals with high neuroticism traits.